# The Gcn5 lysine acetyltransferase mediates cell wall remodeling, antifungal drug resistance, and virulence of *Candida auris*

**DOI:** 10.1128/msphere.00069-25

**Published:** 2025-03-11

**Authors:** Manju Chauhan, Raju Shivarathri, Ariel A. Aptekmann, Anuradha Chowdhary, Karl Kuchler, Jigar V. Desai, Neeraj Chauhan

**Affiliations:** 1 Center for Discovery and Innovation, Hackensack Meridian Health, Nutley, New Jersey, USA; 2 Medical Mycology Unit, Department of Medical Mycology, Vallabhbhai Patel Chest Institute, University of Delhi, Delhi, India; 3 Medical University of Vienna, Max Perutz Labs Vienna, Department of Medical Biochemistry, Campus Vienna Biocenter, Vienna, Austria; University of Georgia, Athens, Georgia, USA

**Keywords:** *Candida auris*, GCN5, lysine acetyl transferase, virulence, caspofungin, CPTH2

## Abstract

*Candida auris* has emerged as a multidrug-resistant human fungal pathogen that causes infections of high morbidity and mortality. However, the molecular mechanisms underlying pronounced multidrug resistance and host–pathogen interactions are poorly understood. Here, we show that *C. auris GCN5* lysine acetyltransferase is essential for cell wall remodeling, antifungal drug resistance, and virulence. The *Candida albicans GCN5* has previously been shown to be an important regulator of antifungal drug resistance and virulence. Therefore, to identify Gcn5-dependent evolutionary conserved as well as divergent transcriptional networks between the two species, we performed comparative transcriptional analysis. The gene set enrichment analysis of *C. auris* vs *C. albicans gcn5*Δ transcriptomic data revealed several major biological pathways and processes including sphingolipid metabolism and glycosylphosphatidylinositol anchor biosynthesis to be enriched in both species. Consistent with these data, we found a prominent role for *C. auris* Gcn5 in maintaining cell–wall architecture, as the *C. auris gcn5*Δ mutant demonstrated a significant increase in cell-surface β-glucan exposure and chitin content. Additionally, we observed that Gcn5 modulates susceptibility to caspofungin and was required for fungal survival when challenged with primary murine macrophages and neutrophils *ex vivo*. Furthermore, disruption of *GCN5* causes virulence attenuation in a murine model of disseminated candidiasis. Lastly, lysine acetyltransferase inhibitor cyclopentanone, 2-[4-(4-chlorophenyl)-2-thiazolyl] hydrazone displayed antifungal activity either alone or in combination with caspofungin against the drug-resistant *C. auris* wild-type strain. Collectively, these data provide new insights into the mechanisms of antifungal drug resistance and *C. auris*–host interactions and suggest Gcn5 lysine acetyltransferase as a potential target for antifungal therapy.

**IMPORTANCE:**

Invasive fungal diseases affect approximately 6.5 million people every year, of which about 2.5 million people die worldwide. This number is expected to rise due to increasing numbers of immunosuppressed people, including the elderly, premature infants, organ transplant recipients, cancer, and HIV/AIDS patients. The Centers for Disease Control and Prevention (CDC) and the World Health Organization (WHO) have both recently emphasized a critical need for the development of new antifungal therapeutics to address expanding drug resistance among human fungal pathogens. The necessity of new antifungal drugs is also underscored by the fact that mortality due to invasive candidiasis has remained unchanged for several decades. However, the discovery of new drugs acting on antifungal drug targets is complicated because fungi are eukaryotes. This greatly limits the number of feasible fungal-specific drug targets. One class of molecules that fulfills the criterion of fungal specificity is chromatin modification enzymes such as lysine acetyltransferase (KATs). The fungal KATs are structurally less well conserved, and some modifications are only found in fungi, minimizing the risk of toxicity, thus making KATs new promising tools for antifungal therapy. We report here that Gcn5 lysine acetyltransferase mediates antifungal drug resistance and virulence of *C. auris* and represents an important target for antifungal drug discovery.

## OBSERVATION

*Candida auris* is a World Health Organization (WHO) fungal priority pathogen (1). The Centers for Disease Control and Prevention (CDC) has classified *C. auris* as an urgent threat to human health due to its clinical and economic impact, high transmissibility, lack of effective antifungal treatments, as well as future projections of new infections over the next 10 years (2). However, despite its profound clinical relevance, our understanding of *C. auris* pathophysiology and virulence, its response to human immune surveillance and underlying host–pathogen interactions, or the molecular bases of multidrug resistance, is poorly understood.

**Fig 1:**
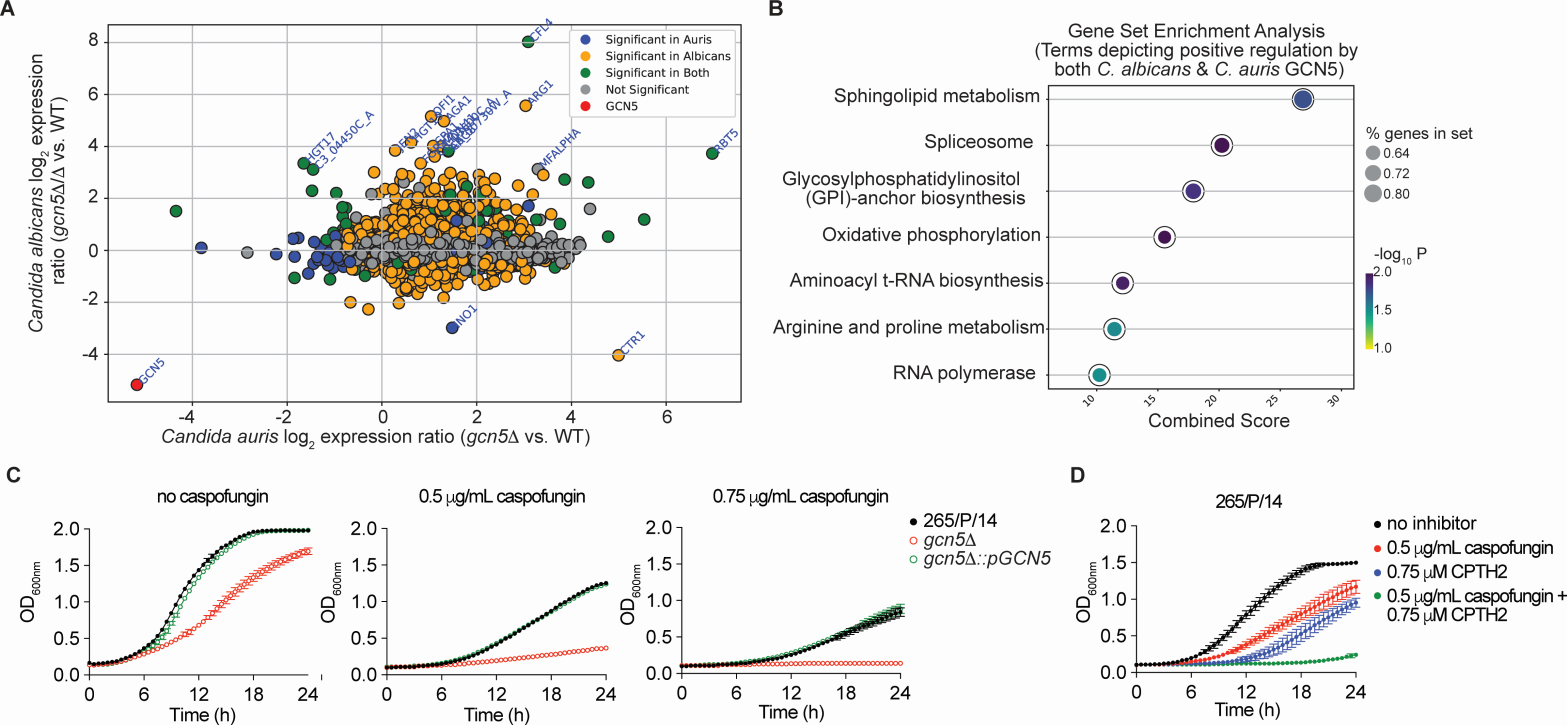
(**A**) Scatter plot showing the log_2_ expression ratios (*gcn5*Δ vs WT) of orthologous genes in *Candida auris* (*x*-axis) and *Candida albicans* (*y*-axis). Each dot represents an orthologous gene. Points are color-coded based on significance: genes significantly upregulated or downregulated in *C. auris* (blue), *C. albicans* (orange), or both species (green). Genes that are not significant in either species are shown in gray, and the *GCN5* gene is marked in red. Highlighted genes display extreme values (>5 std) or biological relevance, as indicated by their labeled names. (**B**) GSEA identifying pathways positively regulated in *gcn5*Δ in both *C. auris* and *C. albicans*. Pathways are ranked by the combined enrichment score (*x*-axis). The dot size represents the proportion of genes in the pathway, and the dot color indicates the –log_10_ adjusted *P*-value, where darker colors represent higher significance. Notable pathways include sphingolipid metabolism, spliceosome, and GPI-anchor biosynthesis. (**C and D**) Growth of indicated strains at 30°C in YPD broth medium containing different concentrations of caspofungin and CPTH2 either alone or in combination with caspofungin and CPTH2. Data represent the mean from three independent experiments.

*GCN5* encodes a lysine acetyltransferase (KAT) that is known to associate with Ada2 and Ada3 to form the catalytic module of the ADA and SAGA transcriptional coactivator complexes, thus forming a dual-layer network of chromatin-mediated transcriptional control (3, 4). Importantly, Gcn5 is essential for resistance to echinocandins and is required for virulence in *Candida albicans* (5, 6). Hence, we hypothesized that Gcn5 similarly promotes echinocandin resistance and virulence of *Candida auris*. To this end, we constructed gene deletion and gene-reconstituted strains in an echinocandin-resistant *C. auris* strain (265 /P/14) belonging to clade I (7, 8) (Text S1). First, to assess the Gcn5-dependent evolutionary conserved as well as divergent transcriptional networks between *C. albicans* and *C. auris,* we performed the comparative RNA-seq profiling of Gcn5-deficient *C. auris* and *C. albicans* strains relative to their wild-type parent. Prior to RNA isolation, all strains were grown to the logarithmic growth phase in YPD broth at 30°C. Differentially expressed genes were defined by a twofold change (log_2_FC 1), with an adjusted *P* value cutoff ≤0.05. Genes were categorized based on their enrichment or depletion across both species (Fig. 1A). To ensure robust cross-species comparisons, we constructed gene sets focusing exclusively on genes that can be mapped between the two species from the KEGG 2019 database and Candida Genome Database (9, 10). The *GCN5*-dependent comparative transcriptomic analysis between *C. auris* and *C. albicans* showed that pathways such as sphingolipid metabolism and GPI anchor biosynthesis are significantly enriched in both species in a *GCN5*-dependent manner (Fig. 1B), suggesting a degree of conservation at the level of transcription in both species. The complete list of differentially expressed genes in the *gcn5*Δ mutant strain is presented in Table S2. Notably, genes belonging to galactose metabolism and ribosome biogenesis showed distinct *C. auris* or *C. albicans* Gcn5-restricted regulation patterns (data not shown). While the species-specific distinct Gcn5-regulated transcripts can be biologically meaningful, based on a significant degree of conservation at the level of transcription in both species, a Gcn5-mediated functional conservation between the two species is likely. The *C. auris* raw RNA-seq data set is available at the NCBI Sequence Read Archive (SRA) collection (PRJNA1232830). The *C. albicans* data set was previously published and is available at NCBI Gene Expression Omnibus (GEO) (GSE123412).

The *C. albicans* Gcn5 has functions in resistance to cell wall stressors, echinocandin (e.g. caspofungin), and promoting virulence (6). Thus, based on our observations of a significant degree of Gcn5-dependent transcriptional conservation in both species, we reasoned for a functional conservation of *C. auris* Gcn5 in caspofungin resistance. Notably, Gcn5 deficiency enhanced the caspofungin susceptibility of the parental isolate (Fig. 1C). The above phenotypes were specific to Gcn5 deficiency as the complementation with a *GCN5* allele restored the parental phenotypes (Fig. 1C). Deletion of *GCN5* also causes a slight reduction in growth rate *in vitro* (Fig. 1C). In this regard, the *C. auris gcn5*Δ is quite similar to *C. albicans gcn5*Δ/Δ, which also shows growth defect *in vitro* (6). Since caspofungin is known to inhibit β-1,3 glucan synthesis in an Fks1-dependent manner, we looked at the expression of *FKS1* and *FKS2* genes in *C. auris* gcn*5*Δ mutant. We noted a significant decrease in the expression of *FKS1* in the *gcn5*Δ compared to the wild-type parental strain. Interestingly, there was a significant increase in the expression of *FKS2* in the *gcn5*Δ mutant, suggesting a compensatory inverse regulation of *FKS* genes by *C. auris* Gcn5 (Table S2).

As caspofungin resistance of *C. auris* poses a significant clinical threat (11–13), based on our data depicting a role for Gcn5 in mediating the caspofungin resistance, we reasoned that the pharmacological targeting of Gcn5 would enhance the caspofungin sensitivity in an otherwise resistant isolate. Furthermore, since fungal Gcn5 recognizes a different lysine configuration on histones compared to its mammalian orthologues (14), it is likely that fungal Gcn5 KAT inhibitors will likely not affect mammalian Gcn5 orthologues. There are currently no antifungal drugs that target KAT function; however, evidence suggests that KATs are potential antifungal targets, despite being non-essential genes (14). Of note, antifungal azoles and echinocandins also target non-essential genes, such as *ERG11* and *FKS1* (demonstrated by generating deletion strains lacking *ERG11* and *FKS1*) (15–18). To explore the role of Gcn5 as a potential target for antifungal drug discovery, we tested the antifungal activity of cyclopentanone, 2-[4-(4-chlorophenyl)-2-thiazolyl] hydrazone (CPTH2) against a wild-type parental isolate exhibiting an enhanced capofungin resistance (Fig. 1C). CPTH2 is a known KAT inhibitor (14, 19). Our results indicate a potent inhibitory effect of CPTH2, equivalent to the level of caspofungin (Fig. 1D). Interestingly, in agreement with our genetic studies, CPTH2-mediated inhibition was synergistic with caspofungin in its antifungal activity, as the *C. auris* exhibited minimal to no growth when grown in the presence of CPTH2 and caspofungin (Fig. 1D).

**Fig 2:**
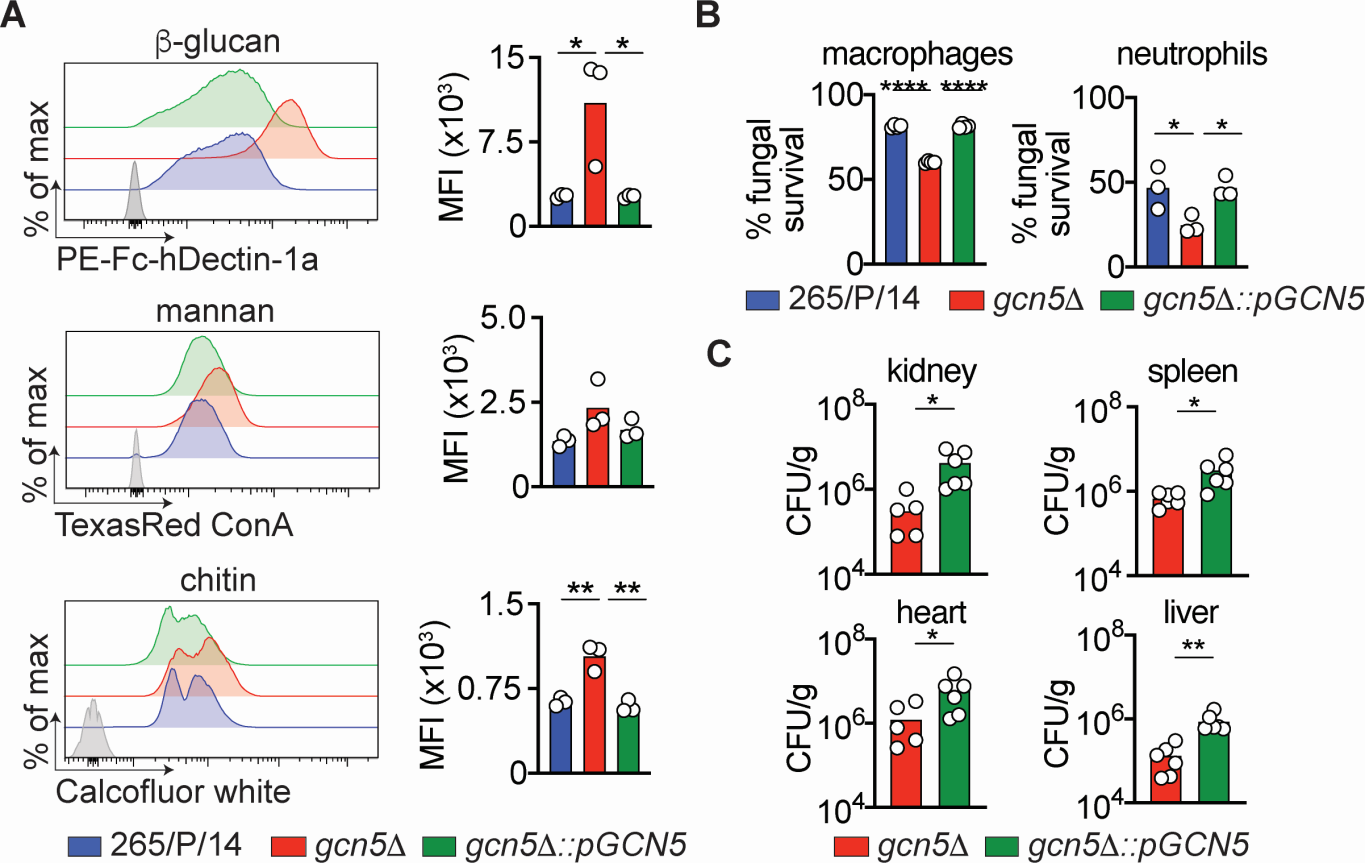
(**A**) *C. auris* strains grown in YPD broth growth media at 30°C were washed and triple stained to quantify cell wall components. Representative flow cytometry histograms and quantification data showing relative β-glucan, chitin, and mannan content in *C. auris* strains. The quantification data shown beside the histograms are from three biological replicates. Dots represent an individual replicate. Error bars represent mean ± SD; *P <  0.05 and **P  < 0.01 by one-way ANOVA with Bonferroni’s multiple comparisons test. (**B**) Percent survival of *C. auris* strains post-challenge with bone marrow-derived macrophages and neutrophils. Data represent the mean of three biological replicates (horizontal line). Dots represent an individual replicate. *P < 0.05 and ****P < 0.0001 by one-way ANOVA with Bonferroni’s multiple comparisons test. (**C**) Fungal burden, at 72 h post-infection for the indicated organs, after systemic infection of the immunocompetent C57/BL6 mice with 5 × 10^7^ *C. auris* CFUs. Data represent the mean of six individual mice. Dots represent individual mice. Error bars represent mean ± SEM; **P <  0.01, by unpaired *t*-test (kidney, spleen, heart) or Mann–Whitney *U* test (liver).

The fungal cell wall, which contains several pathogen-associated molecular patterns (PAMPs), is the first to come in contact with the host phagocytic cells via interactions with the pattern recognition receptors (20). Hence, we then assessed the role of Gcn5 in regulating the fungal PAMPs, with potential implications for *C. auris* interactions with the host phagocytes. To this end, we quantified the major cell wall components such as β-glucans, mannans, and chitin using a flow cytometry-based triple staining assay (21) (Text S1). Interestingly, we observed an increase in β-glucan and chitin in the *gcn5*Δ compared to the wild-type parent and the complemented strains (Fig. 2A). No significant differences were observed in the mannan content between the wild-type parent, *gcn5*Δ, and complemented strains (Fig. 2A). Based on these observations, we hypothesized that Gcn5 is critical for *C. auris*–host cell interactions. Thus, we characterized the *gcn5*Δ strain for its ability to counter the phagocyte-mediated killing and compared it to the wild-type parent and the complemented strains. To this end, we infected murine bone marrow-derived macrophages and neutrophils with wild-type, *gcn5*Δ, and complemented strains at a multiplicity of infection of 5 and co-cultured them for 2.5 h (Text S1). Subsequently, after lysing the phagocytes, the surviving fungal cells were quantified by measuring metabolic activity via a colorimetric assay (22). Strikingly, *gcn5*Δ was much more sensitive to killing by macrophages and neutrophils when compared to wild-type cells (Fig. 2B).

As the *C. auris gcn5*Δ mutant showed reduced fitness when challenged with primary macrophages and neutrophils *ex vivo*, we reasoned that Gcn5 impacts *C. auris* virulence *in vivo*. Thus, we infected immunocompetent C57BL/6 mice (six each of both genders) with *gcn5*Δ and complemented strains systemically with 5 × 10^7^ *C. auris* CFUs and measured fungal burdens in kidneys, spleen, liver, and heart at 72 h post-infection (Text S1). We observed enhanced clearance of *C. auris* in the absence of Gcn5, as the mice infected with the *gcn5*Δ showed significantly reduced fungal burden in the kidneys, spleen, liver, and heart (Fig. 2C). Together, our data indicate that genetic ablation of *C. auris GCN5* abrogates fungal virulence in a mouse model of systemic candidiasis. Furthermore, as noted above, the comparative transcriptomics between *C. albicans* and *C. auris* reveal that Gcn5 modulates transcriptional networks affecting sphingolipid metabolism and GPI anchor biosynthesis (Fig. 1B). Interestingly, the sphingolipid metabolism pathway is an important regulator of echinocandin resistance and fungal virulence (23–25). GPI-anchor cell wall proteins are important mediators of the adhesion of fungal cells to abiotic and biotic surfaces (26). These observations provide support to the hypothesis that cell wall architecture, caspofungin resistance, and virulence are likely governed, in part, by Gcn5-dependent transcriptional networks in both species. In future studies, it will be of interest to characterize the Gcn5-dependent sphingolipid metabolism and GPI-anchored cell surface genes for their role in antifungal drug resistance and host–pathogen interactions.

In conclusion, the data presented herein highlight the relative importance of Gcn5 lysine acetyltransferase in antifungal drug resistance and host*–C. auris* interactions and suggest its exploitation could lead to the development of drugs that may be broad spectrum.

## Data Availability

The *C. auris* raw RNA-seq data set is available at the NCBI Sequence Read Archive (SRA) collection (PRJNA1232830).

## SUPPLEMENTAL MATERIAL

Supplemental material msphere.00069-25-s0001.docx Detailed methods and supplemental tables.

Table S2 msphere.00069-25-s0002.xlsx Differentially expressed gene list.

ASM does not own the copyrights to Supplemental Material that may be linked to, or accessed through, an article. The authors have granted ASM a non-exclusive, world-wide license to publish the Supplemental Material files. Please contact the corresponding author directly for reuse.
