# Supplementary material for: The Gcn5 lysine acetyltransferase mediates cell wall remodeling, antifungal drug resistance, and virulence of Candida auris
Source: mSphere. 2025 Mar 11;10(4):e00069-25. doi: 10.1128/msphere.00069-25 (PMC12039264; doi:10.1128/msphere.00069-25)
Supplement: Supplemental material — Detailed methods and supplemental tables. [file msphere.00069-25-s0001.docx]

**Supplementary Text S1.**

***Candida auris* strains, media, and growth conditions**

The *Candida auris* strains 265/P/14, *gcn5*Δ and *gcn5*Δ::GCN5 used in this study are listed in Table S1. All *C. auris* strains were routinely grown on YPD medium (1% yeast extract, 2% peptone, and 2% glucose (BD Biosciences) at 30°C with or without shaking at 200 rpm. For solid medium, 2% Bacto agar (BD Biosciences) was added to YPD broth. Nourseothricin and Hygromycin at 200 μg/ml were used as selection markers for *C. auris*.

**Plasmid and *C. auris* *GCN5* deletion strain construction**

The deletion of *GCN5* (B9J08_005133) was performed by using the modified fusion PCR method (1). Briefly, upstream and downstream flanking regions of the *GCN5* open reading frame (ORF) were PCR amplified using primers 53_CauGCN5 and 35_CauGCN5. These primers include an additional 20-bp constant overlap sequences U1 and D1 at the 3′ end of upstream and 5′ end of downstream regions, respectively. All PCR primers are listed in Table S2. The dominant marker NAT1 was amplified from the plasmid pSFS3b (2) using the primers NAT1_fwd_U1 and NAT1_rev_D1 covering constant complementary 20-bp sequences U1 and D1. The PCR-amplified upstream, downstream, and NAT1 marker fragments were gel purified and subjected to a fusion PCR reaction using Ex Taq polymerase (Takara). The conditions used for the fusion PCR were as follows: for 50-μl reaction volume, 1× Ex Taq buffer, 0.2 μM deoxynucleoside triphosphates (dNTPs), 0.5 μM (each) primer, 3 μl marker fragment, 1.25 μl each flanking homology fragment, and 0.25 μl Ex Taq polymerase; 98°C for 5 min; 30 cycles of 98°C for 20 s, 50°C for 30 s, and 72°C for 1 min (for 1-kb fragment); and a final extension at 72°C for 10 min. The purified gene deletion constructs were used to transform into *C. auris* clinical isolate 265/P/14. Transformation of *C. auris* was done via the lithium acetate (LiAc)/single-stranded (SS) carrier DNA/polyethylene glycol (PEG) method as described previously (1). Strains were initially verified by colony PCR and then later genomic DNA PCR to confirm correct genomic integration of the deletion cassette, as well as the loss of the coding sequence. The Gcn5-gene reconstituted strain was constructed by using the infusion cloning approach. Briefly, the plasmid pYM70 containing the hygromycin resistance marker (3) was PCR amplified by using the primers NC588_pYM70_Fwd: and NC589_pYM70_Rev. Next, the coding sequence of *C. auris* *GCN5* along with its 5′ UTR and 3′ UTR (~500 bp each) was PCR amplified from the parental strain (265/P/14) genomic DNA using the following primers NC592_CauGCN5int_F and NC593_CauGCN5int_R. Both primers contain the overhang sequences (in lowercase) complementary to the plasmid pYM70. The resulting PCR products were fused together using the Infusion Cloning Kit (Takara). The recombinant plasmid thus created was linearized by using SacII and transformed into the *gcn5*Δ strain to reintegrate the *GCN5* ORF at its native locus. The correct integration of *GCN5* ORF into the gene-reconstituted strains was confirmed by PCR.

**Growth and phenotypic assays**

To study the effect of in vitro stressors and antifungal drugs, the *C. auris* strains were grown in YPD broth overnight at 30°C. From an overnight culture, cells corresponding to an optical density at 600 nm (OD_600_) of 0.1 were inoculated into fresh YPD broth with or without caspofungin, amphotericin B, fluconazole, calcofluor white, caffeine, SDS, anacardic acid and CPTH2. Absorbance was recorded in a Tecan Spark plate reader at regular intervals for a period of 24 h, and the OD_600_ values were plotted versus time.

#### **RNA Sequencing and Quality Control**

For transcriptional profiling experiments, the *C. auris* wildtype (265/P/14) and *gcn5*Δ strains were grown to logarithmic growth phase in YPD broth at 30°C. Total RNA from 3 independent biological replicates from each strain was purified using the TRI reagent (Sigma). RNA sequencing was performed using the Illumina HiSeq2000 platform. Paired end reads of 150 bp were generated. Raw reads were assessed for quality using **FastQC** 0.12.1b. Low-quality bases and adapter sequences were trimmed using **Trimmomatic** 0.39.

#### Transcript Alignment and Quantification

Cleaned reads were aligned to the reference genome *Candida auris* B11243 from ENSEMBLE using **STAR** 2.7.11b. Alignment was assessed using **SAMtools** 1.21 and **Picard** 3.0 to calculate metrics such as mapping percentage and insert size distribution.

#### Differential Expression Analysis

Differential expression analysis was conducted using **DESeq2** 1.47.1 in the R studio environment version 2024.12.0.467 with R v 3.6.1. Raw read counts were normalized using the median of ratios method to account for differences in sequencing depth and library size. Experimental design included terms for species and genetic background to derive species-specific as well as conserved *GCN5*-dependent transcriptional changes. Gene-wise dispersion estimates were computed, and statistical tests were performed to identify differentially expressed genes (DEGs). Genes with an adjusted p-value < 0.05 (Benjamini-Hochberg) and a |log2 fold-change| > 1] were considered significantly differentially expressed.

#### Functional Enrichment Analysis

Gene set enrichment analysis (GSEA) was performed using **GSEAPy**, a Python-based implementation of GSEA. Normalized expression data were ranked based on the signed log-transformed p-values from DESeq2. GSEA was conducted against GO and KEGG gene sets. Enrichment scores were calculated using weighted Kolmogorov-Smirnov statistic, and significance assessed using 1,000 permutations of gene labels. Gene sets with a false discovery rate (FDR) < 0.05 were considered significantly enriched.

#### Data Visualization and Availability

Visualizations of differential expression results, including volcano plots and heatmaps, were generated using **ggplot2.** Enrichment results were visualized using dot plots and bar charts in **Matplotlib** and **Seaborn**. The *C. auris* raw dataset is available at NCBI SRA collection (SUB13802269). The *C. albicans* dataset was previously published and is available at NCBI SRA collection (GSE123412).

**Cell wall quantification assay**

The cell wall components were quantified by using a flow cytometry-based approach as described previously (3). Briefly, the *C. auris* strains were grown to logarithmic growth phase in YPD broth at 30^0^C. The logarithmically growing cultures were washed and stained with concanavalin A-conjugated Texas Red, Fc-hDectin-1a, and calcofluor white to quantify the mannans, glucan, and chitin, respectively. These triple-stained cells were measured in a BD Fortessa flow cytometer (BD Biosciences) to quantify the amount of chitin, glucan, and mannan using the BV421 (violet 405 nm, 50-mW power), fluorescein isothiocyanate (FITC) (blue 488-nm wavelength, 50-mW power), and Texas Red (red 640-nm wavelength, 40-mW power) detectors, respectively. A minimum of 10,000 events were recorded for each sample, and the data were analyzed using FlowJo software (BD Biosciences). Unstained and single-stained samples served as controls, and the data were expressed as the mean fluorescence intensity (MFI) from three independent experiments.

**Macrophage/neutrophil killing assay**

Primary cultures of bone marrow-derived macrophages (BMDMs) were isolated and cultivated exactly as described before (3). Neutrophils were isolated using a mouse neutrophil isolation kit (Miltenyi Biotec) according to manufacturer instructions. Survival of *C*. *auris* in BMDMs and neutrophils was quantified as described previously (3) using an MOI of 5:1 (fungi to macrophages or neutrophils). Fungal cells were harvested 2.5 hours post-infection by lysing the neutrophils/BMDMs with cold water containing 0.1% Triton-X followed by two washes with water. Next, 100 μl of XTT (0.4 mg/mL 2,3-bis-(2-methoxy-4-nitro-5-sulfophenyl)-2H-tetrazolium-5-carboxanilide) and coenzyme Q_0_ (0.05 mg/mL 2,3-dimethoxy-5-methyl-p-benzoquinone) reagent mix was added to the lysates and incubated at 37°C for 1 hour. Finally, absorbance at 450 nm with 660 nm for background correction was measured. Survival was calculated as a percentage of viable CFUs by comparing with uninfected *Candida* strains.

**Murine model systemic candidiasis and fungal burden determination**

All animal experiments were performed under the auspices of the approved protocols 293 and 298 by the Center of Discovery and Innovation (CDI) Institutional Animal Care and Use Committee (IACUC). For all animal experiments, 8- to 12-week-old C57BL/6 (Jackson Laboratory) wildtype female mice were used. The infections were performed as described previously (3), with minor modifications. Prior to infection with *C. auris,* the mice were anesthetized using xylazine/ketamine cocktail. The mice were infected with 5x10^7^ *C. auris* CFUs retro-orbitally. At 72h post infection, the mice were sacrificed to quantify fungal burdens from infected kidneys, liver, spleen and heart. The fungal burdens were quantified by plating the homogenates on YPD agar plates containing penicillin and streptomycin, and enumerating the CFUs after 48h of growth at 30°C.

**Statistical Analyses**

To determine statistical significance across groups, we used two-sided unpaired *t* tests (with or without Welch’s correction, as appropriate), two-sided paired *t* tests, two-sided Mann-Whitney *U* tests, or ordinary one-way ANOVA with Tukey’s post hoc multiple-comparison test, as appropriate, using GraphPad Prism 9 software (GraphPad Software, LLC). A *P* value of less-than or equal-to 0.05 was considered significant.

**Table S1. List of *Candida auris* strains used in the current study**

| Stain name | Genotype | Reference |
| --- | --- | --- |
| 265/P/14 | Clade I echinocandin resistant C. auris clinical isolate | (4, 5) |
| *gcn5*Δ | *gcn5*Δ::*NAT1* | This study |
| *gcn5*Δ::*GCN5* | *gcn5*Δ::*GCN5*::*NAT1*,*HYG1* | This study |

**Table S3. List of primers used in the current study**

| Number | Primer name | Primer Sequence (5’-3’) |
| --- | --- | --- |
| 1 | 53_CauGCN5 | gtcagcggccgcatccctgcGAGGCTCTGGTGAAGTTTAC |
| 2 | 35_CauGCN5 | cacggcgcgcctagcagcggGCAACTAACGGTTTTCATGG |
| 3 | NAT1_U1_F | ccgctgctaggcgcgccgtgAGCTTGCCTCGTCCCCGCCG |
| 4 | NAT1_D1_R | gcagggatgcggccgctgacCTGGATGGCGGCGTTAGTATCG |
| 5 | Int1_CauGCN5_F | GACGGGGTAGAGTACTCCTT |
| 6 | Int1_CauGCN5_R | TGCATTAGAGTACCACCTTC |
| 7 | 588_pYM70_Fwd | GTATAGTGCTTGCTGTTCGATATTG |
| 8 | 589_pYM70_Rev | GACGTCAGGTGGCACTTTTCG |
| 9 | 592_CauGCN5int_F | gtgccacctgacgtcGACGGCTTCTGAACACTGAGC |
| 10 | 593_CauGCN5int_R | cagcaagcactatacGGCATTATCAGAGCCTTCAGTTTG |

**Table S4. List of plasmids used in the current study**

| Number | Plasmid name | Reference |
| --- | --- | --- |
| 1 | pSFS3b | (2) |
| 2 | pYM70 | (3) |

**Supplementary Material References**

1. Shivarathri R, Jenull S, Stoiber A, Chauhan M, Mazumdar R, Singh A, Nogueira F, Kuchler K, Chowdhary A, Chauhan N. 2020. The Two-Component Response Regulator Ssk1 and the Mitogen-Activated Protein Kinase Hog1 Control Antifungal Drug Resistance and Cell Wall Architecture of Candida auris. mSphere 5.

2. Shivarathri R, Tscherner M, Zwolanek F, Singh NK, Chauhan N, Kuchler K. 2019. The Fungal Histone Acetyl Transferase Gcn5 Controls Virulence of the Human Pathogen Candida albicans through Multiple Pathways. Sci Rep 9:9445.

3. Shivarathri R, Chauhan M, Datta A, Das D, Karuli A, Aptekmann A, Jenull S, Kuchler K, Thangamani S, Chowdhary A, Desai JV, Chauhan N. 2024. The Candida auris Hog1 MAP kinase is essential for the colonization of murine skin and intradermal persistence. mBio 15:e0274824.

4. Fakhim H, Chowdhary A, Prakash A, Vaezi A, Dannaoui E, Meis JF, Badali H. 2017. In Vitro Interactions of Echinocandins with Triazoles against Multidrug-Resistant Candida auris. Antimicrob Agents Chemother 61.

5. Kordalewska M, Lee A, Park S, Berrio I, Chowdhary A, Zhao Y, Perlin DS. 2018. Understanding Echinocandin Resistance in the Emerging Pathogen Candida auris. Antimicrob Agents Chemother 62.
